# Preoperative Clinical Predictors of Histologic Malignancy and Carcinoma Grade in 286 Canine Mammary Nodules from 92 Bitches: A Retrospective Study

**DOI:** 10.3390/ani16030421

**Published:** 2026-01-29

**Authors:** Manuel Fuertes-Recuero, Paula García San José, Guillermo Valdivia, María Suarez-Redondo, Silvia Penelo, Mario Arenillas, Laura Camacho-Alonso, Laura Peña, Dolores Pérez-Alenza, Gustavo Ortiz-Díez

**Affiliations:** 1 Complutense Veterinary Teaching Hospital, Complutense University of Madrid, Avda. Puerta de Hierro s/n, 28040 Madrid, Spain; manufuer@ucm.es (M.F.-R.); pgsanjose@ucm.es (P.G.S.J.); edgargva@ucm.es (G.V.); marsuare@ucm.es (M.S.-R.); spenelo@ucm.es (S.P.); marioare@ucm.es (M.A.); laurcama@ucm.es (L.C.-A.); laurape@vet.ucm.es (L.P.); gusortiz@ucm.es (G.O.-D.); 2 Department of Physiology, Veterinary Medicine School, Complutense University of Madrid, Avda. Puerta de Hierro s/n, 28040 Madrid, Spain; 3 Department of Animal Medicine and Surgery, Veterinary Medicine School, Complutense University of Madrid, Avda. Puerta de Hierro s/n, 28040 Madrid, Spain

**Keywords:** canine mammary tumours, prediction model, histological malignancy, histological grade, preoperative clinical predictors, firth logistic regression, clustered data

## Abstract

Canine mammary tumours often present as multiple synchronous nodules, necessitating decisions regarding staging intensity and surgical planning prior to histology. We developed two preoperative nodule-level prediction models using only the medical history and physical examination of client-owned bitches with mammary disease, which were staged using the WHO-modified TNM system with a M0 classification (no distant metastasis) at the time of presentation. This retrospective study analysed 286 surgically excised mammary nodules from 92 dogs managed under a standardised mammary oncology protocol; those with inflammatory mammary carcinoma or distant metastasis were excluded. The outcomes were (i) malignant versus benign/non-neoplastic histology (for all nodules) and (ii) intermediate/high histologic grade (II–III versus I) among carcinomas. Separate multivariable Firth penalised logistic regression models accounted for within-dog clustering with dog-level bootstrap internal validation. Multiple imputation was used in a sensitivity analysis for missingness in the detection-to-surgery interval. Malignancy was confirmed in 87/286 (30.4%) of the nodules (86 carcinomas), including 35/87 (40.2%) that measured less than 1 cm. Among complete cases (153 nodules), malignancy was associated with age at neutering, maximum tumour diameter, owner-reported rapid growth and a detection-to-surgery interval of more than 3.5 months (an exploratory ROC-derived threshold) with good discrimination (area under the curve (AUC) 0.805; optimism-corrected 0.799) and acceptable calibration. Among carcinomas (83 specimen), previous mammary tumours, bloody nipple discharge and fewer synchronous nodules were associated with intermediate/high malignancy grade (AUC 0.859). Sensitivity analyses yielded directionally consistent estimates. Routinely available clinical information may provide interpretable preoperative risk stratification to support staging and surgical planning, pending external validation.

## Simple Summary

Canine mammary tumours are common in female dogs, and many have more than one nodule. During the initial consultation, veterinarians must determine which tests are necessary to evaluate whether the disease has spread (staging) and the extent of the required surgery. However, the malignancy of a nodule and its histological aggressiveness can only be determined after its removal and pathological examination. We reviewed the records of 92 client-owned female dogs with no evidence of distant metastasis at the time of presentation, including 286 surgically removed mammary nodules. Using statistical methods that accounted for multiple nodules from the same dog, we found that malignant nodules were more prevalent among those that were larger, had reportedly grown quickly, or for which a longer interval had been observed between initial detection and surgery. However, it should be noted that small nodules can still be malignant. Among mammary carcinomas, higher-grade tumours were more prevalent in dogs with a history of mammary tumours, bloody nipple discharge and fewer nodules detected at the same time. These everyday clinical findings may help clinicians prioritise staging tests, support surgical planning and counsel owners while awaiting the definitive pathology report. However, the prediction models should be confirmed in other hospital populations before they can be used more widely.

## 1. Introduction

Canine mammary tumours (CMTs) are among the most common neoplasms in female dogs, particularly in intact bitches, and account for a significant proportion of tumours in this population. Approximately 40–60% of these tumours are malignant [1,2,3,4]. Malignant CMTs substantially contribute to cancer-related mortality in female dogs [5,6,7,8]. Although heterogeneous, CMTs share clinicopathological features with certain human breast cancer subtypes, supporting their use as a comparative model in selected translational contexts [1,2,6,9]. Despite their relevance, predicting whether a mammary mass is malignant and anticipating histologic aggressiveness preoperatively using only information available at first presentation remains a major clinical challenge.

Disease extent is routinely assessed using the WHO-modified TNM system, which categorises primary tumour size (T), regional lymph node status (N) and distant metastasis (M), and stages I–V are defined with prognostic relevance [10]. Clinical stage and readily obtainable tumour and patient characteristics, most notably larger tumour size, older age, and ulceration, have been associated with a higher likelihood of histological malignancy and aggressive behaviour [4,11]. However, these factors do not reliably enable nodule-level preoperative discrimination between benign/non-neoplastic and malignant lesions nor do they permit preoperative determination of histologic grade [2,6,9]. This limitation is particularly relevant, because many dogs present with multiple synchronous mammary nodules and different nodules within the same dog may differ in histology and grade [2,6,9].

In practice, dogs often present with one or more mammary masses that vary in size and appearance. Multifocal disease is also common at diagnosis [1]. Preoperative assessment typically relies on physical examination, fine-needle aspiration cytology and standard imaging; however, these tools may not reliably distinguish between benign and malignant lesions at the nodule level. B-mode and Doppler ultrasonography generally demonstrate moderate diagnostic performance. Advanced techniques such as acoustic radiation force impulse (ARFI) elastography have demonstrated promising diagnostic performance in limited studies. Nevertheless, a definitive diagnosis still relies on postoperative histopathology [12,13]. Importantly, histologic grade, one of the strongest prognostic indicators [14,15], is only available after biopsy or excision.

Although extensive and increasingly multivariate prognostic research has been conducted on CMTs, most published models incorporate postoperative histological and/or immunohistochemical variables [14,16]. In contrast, clinically usable preoperative models based on readily available medical history and physical examination data are limited, and the histologic grade is rarely evaluated as a dedicated preoperative prediction endpoint. The simultaneous preoperative prediction of histological malignancy and grade remains uncommon. In a large multicentre study, tumour size and age were identified as predictors of histological malignancy, yet the overall accuracy was modest, indicating a need for more robust and clinically applicable models [17]. Additionally, published studies differ in eligibility criteria and stage distribution; some include dogs with distant metastasis (M1) and/or inflammatory mammary carcinoma (IMC), which are clinical entities with distinct biology and clinical management. This variability limits direct comparability across study population and may influence observed associations between preoperative findings and histologic outcomes [5,9].

Accordingly, the present study focused on mammary disease with M0 status at presentation (i.e., no evidence of distant metastasis), and included both local (M0N0) and regional (M0N1) disease. The disease was staged according to the modified WHO TNM system [10]. To ensure a clinically homogeneous study population, dogs with distant metastasis (M1) at presentation and those diagnosed with IMC were excluded [9]. The aim of the study was to investigate which readily obtainable history and physical examination variables at presentation were associated with (1) histologic malignancy (benign/non-neoplastic vs. malignant) and (2) intermediate/high histologic grade (grades II–III) among carcinomas. The objective of the nodule-level analyses, which accounted for within-dog correlation, was to develop practical preoperative prediction models to support risk-stratified staging, surgical planning and owner counselling in everyday practice.

## 2. Materials and Methods

### 2.1. Study Design and Population

This retrospective analytical study included client-owned bitches that underwent the surgical removal of mammary tumours at the Veterinary Teaching Hospital (VTH) of the Complutense University of Madrid (Madrid, Spain) between January 2020 and December 2023. All patients were managed within the hospital’s standardised institutional protocol, which follows a standardised, multidisciplinary clinical protocol encompassing diagnostic work-up, staging, surgical management, histopathology, and follow-up. This structured clinical workflow ensured the systematic collection of preoperative variables at first presentation, strengthening internal consistency despite the retrospective design.

Dogs were eligible if they underwent the surgical excision of one or more mammary nodules during the study period and if the electronic medical record and final histopathology report(s) provided sufficient information to confirm signalment and assign a histologic diagnosis (benign/non-neoplastic vs. malignant) for all excised nodules included in the dataset. The study focused on mammary disease with M0 status at presentation (no evidence of distant metastasis); therefore, dogs with distant metastasis (M1) and those diagnosed with inflammatory mammary carcinoma were excluded a priori, as these presentations represent distinct clinical entities managed under different pathways and fall outside the target population for a preoperative prediction tool intended for surgically managed M0 disease. Ninety-two dogs met these criteria and contributed a total of 286 surgically excised mammary nodules.

The primary unit of analysis was the individual nodule. Because multiple nodules could originate from the same dog, nodule-level analyses explicitly accounted for within-dog correlation, as detailed in Section 2.5. Analyses of histological grade were restricted to mammary carcinomas. Written owner consent for the use of clinical data for teaching and research purposes is routinely obtained at hospital admission.

### 2.2. Clinical Evaluation and Preoperative Data Collection

Preoperative evaluation followed the standardised institutional protocol and included a detailed medical history, complete physical examination with the systematic palpation of both mammary chains and regional lymph nodes, and a caliper measurement of the maximum diameter (cm) of each palpable nodule.

Clinical stage at admission was assigned according to the modified WHO TNM system for canine mammary tumours [10]. The staging work-up routinely comprised three-view thoracic radiography. Abdominal ultrasonography was performed when clinically indicated (high tumour burden, suspected lymph node involvement, or relevant comorbidities) and was therefore not systematic across all dogs. Fine-needle aspiration cytology was not routinely performed on mammary nodules as part of this protocol. The cytologic assessment of regional lymph nodes was obtained selectively when lymphadenomegaly was detected on clinical examination or imaging and when results were considered likely to influence surgical planning. Routine preoperative procedures also included complete blood count and serum biochemistry.

Dogs were classified as having local (M0N0) or regional (M0N1) disease. Regional disease (M0N1) required the cytologic or histologic confirmation of regional nodal metastasis; dogs were considered local (M0N0) in the absence of such evidence. This classification was used for descriptive purposes and does not imply any systematic histologic confirmation of lymph node-negative status in all dogs.

Data were extracted from electronic medical records at two levels: dog level and nodule level. At the dog level, recorded variables included age at surgery, breed, body weight, reproductive status (intact or spayed), age at neutering (for spayed dogs), history of pseudopregnancy, history of previous mammary tumours and their histology when available, previous litters, relevant systemic comorbidities, prior hormonal treatments (progestogens or cabergoline), other chronic medications, and abnormalities in preoperative haematology and biochemistry. When ovariohysterectomy was performed concurrently with mastectomy, histopathologic findings from the ovaries and uterus (cystic endometrial hyperplasia, pyometra, ovarian cysts, or tumours) were also recorded.

At the nodule level, variables recorded for each surgically excised lesion included anatomical location (mammary gland), maximum diameter (cm) measured preoperatively, the presence and type of nipple discharge (categorised as none/other, serous or milk-like, or bloody), local inflammatory signs (erythema, heat, induration, or pain), ulceration, adhesion to deeper tissues (fascia or muscle), and the clinical assessment of ipsilateral axillary and superficial inguinal lymph nodes (normal or enlarged).

The number of synchronous nodules was defined as the number of mammary nodules detected on the preoperative mammary examination at first presentation. Nodules identified only during surgical exploration or on gross examination of the excised specimen were not included in this count and were not considered preoperative predictors.

The detection-to-surgery interval (months) was defined as the time from owner-reported first detection of any mammary lesion to the date of surgical excision and was assigned to all nodules from the same dog. “Rapid growth” was recorded as an owner-reported history of rapid increase in nodule size at first presentation and coded as a binary variable as captured in the medical record.

### 2.3. Surgical Management and Specimen Handling

The type of surgery (nodulectomy, regional mastectomy, or unilateral radical mastectomy) was selected according to clinical stage and local factors, including tumour size, number and distribution of nodules, and suspected adhesion to deeper tissues, following institutional guidelines and contemporary surgical recommendations. Regional lymph node management followed unit protocols: inguinal lymphadenectomy was routinely performed when inguinal mammary glands were removed, whereas axillary lymph node excision was undertaken when nodes were enlarged or otherwise suspicious based on clinical assessment and/or cytology. Ovariohysterectomy was performed in intact dogs at the time of mastectomy when considered clinically appropriate and authorized by the owner [5].

All excised mammary tissues and lymph nodes were fixed in 10% neutral-buffered formalin and submitted to the pathology service of the VTH, where specimens were processed according to standard histopathological procedures.

### 2.4. Histopathology, Grading, and Outcome Definitions

Tumours were histologically classified according to established international criteria for canine mammary tumours based on routine haematoxylin and eosin-stained sections and current diagnostic guidelines [2,15]. Mammary carcinomas were graded I–III using the Peña three-tier grading system, which evaluates tubule formation, nuclear pleomorphism, and mitotic count and has been validated for prognostic assessment in non-inflammatory canine mammary carcinomas [14]. All histopathological evaluations were performed as part of routine diagnostic practice by board-certified veterinary pathologists.

Two binary outcomes were defined at the nodule level. The first outcome was histological malignancy, which was classified as malignant versus benign/non-neoplastic and evaluated in all 286 excised nodules. Benign/non-neoplastic lesions included benign mammary tumours and non-neoplastic proliferative changes.

The second outcome was histological grade among mammary carcinomas, which was defined as intermediate/high grade (grades II–III) versus low grade (grade I) according to the Peña grading system [14].

Spindle cell mammary tumours, including mammary sarcomas, carcinosarcomas, malignant myoepithelioma, and carcinoma and malignant myoepithelioma, were classified as malignant for the malignancy outcome but were excluded from all analyses involving histological grade. This decision was prespecified and based on current evidence indicating the following: no validated or prognostically reliable grading system exists for mammary sarcomas or carcinosarcomas, and the prognostic value of applying mammary carcinoma grading schemes to malignant myoepithelioma or carcinoma and malignant myoepithelioma remains insufficiently supported [9,15]. Accordingly, grade-based analyses were restricted to mammary carcinomas for which the Peña grading system has demonstrated prognostic validity. In the present study, one malignant myoepithelioma was identified and was included in malignancy analyses but excluded from all grade-based analyses in accordance with these criteria.

### 2.5. Statistical Analysis

Analyses were conducted at the nodule level. Because some dogs contributed multiple nodules, within-dog correlation was addressed using two complementary approaches. For univariable logistic regression, inference used dog-clustered standard errors. For multivariable penalised models, the coefficient uncertainty was obtained using dog-level cluster bootstrap resampling (sampling dogs with replacement and retaining all nodules per selected dog).

Two separate multivariable models were developed: (i) histological malignancy (malignant vs. benign/non-neoplastic) in all nodules; and (ii) histological grade among mammary carcinomas, which was defined as intermediate/high grade (grades II–III) versus low grade (grade I). Candidate predictors were examined in univariable analyses; variables with *p* < 0.20 and covariates considered clinically relevant a priori were considered for multivariable modelling. The detection-to-surgery interval was additionally analysed using an exploratory, data-driven binary threshold (>3.5 months) derived from ROC analysis using Youden’s index (J = sensitivity + specificity − 1) for the malignancy outcome. This cut-off was selected to improve clinical interpretability and was not prospectively prespecified or externally validated; therefore, it should be considered hypothesis-generating rather than a validated clinical decision boundary.

Multivariable analyses were fitted using Firth’s penalised logistic regression to reduce small-sample bias and address quasi-separation. Effects are reported as odds ratios (OR) with 95% confidence intervals (CI). Multicollinearity was assessed using variance inflation factors (VIFs) calculated from the maximum-likelihood equivalent model fitted on the same estimation sample (prespecified threshold < 5). Model discrimination was quantified using the area under the ROC curve (AUC). Calibration was assessed using calibration slope estimates and calibration plots; where applicable, a Hosmer–Lemeshow statistic from the maximum-likelihood equivalent model was reported as an ancillary goodness-of-fit check.

Primary analyses were conducted using complete-case datasets. As a sensitivity analysis for missing data, multiple imputation was performed under a missing-at-random assumption within Stata’s mi framework. Given the monotone missing-data pattern and to ensure model stability, imputation was restricted to the detection-to-surgery interval (delay_mo), which was imputed by linear regression with 40 imputations (m = 40; seed 240,911). The outcome and key predictors were included in the imputation model, and the exploratory binary variable delay_gt35 (>3.5 months) was updated passively after imputation. Sensitivity analyses were fitted using logistic regression with dog-clustered standard errors and combined using Rubin’s rules.

Internal validation was performed using 1000 dog-level bootstrap resamples. Two-sided *p*-values < 0.05 were considered statistically significant. Reporting followed STROBE and TRIPOD recommendations [18,19]. All analyses were performed in Stata 15.0 (StataCorp LLC, College Station, TX, USA).

## 3. Results

### 3.1. Study Population

A total of 286 mammary nodules from 92 bitches were analysed. Histopathology confirmed 87/286 (30.4%) malignant lesions, including 86 carcinomas and one malignant myoepithelioma (classified as malignant for the malignancy outcome but excluded from all grade-based analyses, as prespecified). Mean age at surgery was 10.1 ± 2.6 years (median 9.9; IQR 8.6–12.2), and mean body weight was 14.3 ± 10.6 kg (median 10.1; IQR 5.7–22.0). Among spayed bitches, mean age at neutering was 9.0 ± 2.9 years (median 9.0; IQR 7–11). Age at neutering was missing for 10/92 (10.9%) dogs; among those with available data, neutering at ≤3 years was uncommon (5/82, 6.1%). Most dogs were purebred (67/92, 72.8%). The most frequently represented breeds were Yorkshire Terrier (*n* = 13) and German Shepherd (*n* = 6) with the remainder distributed across multiple breeds.

At presentation, clinical stage was classified as local disease (M0N0) in 81/92 (88.0%) dogs and regional disease (M0N1) in 11/92 (12.0%). On physical examination, inguinal lymphadenopathy was detected in 17/92 (18.5%) dogs and axillary lymphadenopathy in 5/92 (5.4%). Histopathology confirmed inguinal lymph node metastasis in 9/92 (9.8%) dogs, whereas axillary metastasis was not observed. Dog-level characteristics are summarised in Table 1, and nodule-level descriptors are shown in Table 2. At first presentation, 63/92 (68.5%) bitches had ≥2 synchronous nodules (range 1–14; median 2, IQR 1–4). Frequencies were 1 (*n* = 29), 2 (*n* = 22), 3 (*n* = 13), and ≥4 (*n* = 28). Ulceration was recorded in 10/286 (3.5%) nodules, including 5/87 (5.7%) malignant and 5/199 (2.5%) benign/non-neoplastic lesions. 

### 3.2. Tumour Size Distribution

The overall median maximum diameter was 0.6 cm (IQR 0.3–1.6; mean 1.34 ± 1.91 cm). Malignant nodules were larger than benign nodules (median 1.3 cm [IQR 0.5–3.1] vs. 0.5 cm [IQR 0.3–1.0]; *p* < 0.001), yet 35/87 (40.2%) malignant lesions measured <1 cm (Figure S1). Among carcinomas, the maximum diameter was numerically higher in grades II–III than in grade I (median 1.6 cm [IQR 0.8–3.6] vs. 1.0 cm [IQR 0.5–2.3]) with substantial overlap; this difference was not statistically significant (Figure S2; *p* = 0.069).

When the tumour size was categorised according to the modified WHO TNM T component [10], the proportion of malignant lesions increased across T categories (T1–T3; Table S3; Figure S3). Despite this gradient, most malignant nodules were small with 62/87 (71.3%) classified as T1 (<3 cm).

### 3.3. Multivariable Model for Malignancy

The multivariable malignancy model was developed on a complete-case dataset of 153/286 nodules (53.5%), excluding observations with missing data in one or more final predictors. In Firth penalised logistic regression, four preoperative variables were retained in the final model and were associated with histologic malignancy (Table 3): age at neutering (OR 0.76 per year, 95% CI 0.65–0.88; *p* < 0.001), maximum tumour diameter (OR 1.46 per cm, 95% CI 1.17–1.82; *p* = 0.001), owner-reported rapid growth (OR 3.11, 95% CI 1.15–8.41; *p* = 0.025), and a detection-to-surgery interval >3.5 months (exploratory ROC-derived threshold; OR 3.20, 95% CI 1.23–8.27; *p* = 0.017).

Model calibration was acceptable (calibration slope 1.06; intercept 0.03; Hosmer–Lemeshow χ^2^ = 4.68, *p* = 0.79), and discrimination was good (AUC 0.805, 95% CI 0.731–0.880). Internal validation using 1000 dog-level bootstrap resamples yielded an optimism-corrected AUC of 0.799. The full prediction equation, including the intercept and a worked example for clinical use, is provided in Supplementary Box S1.

### 3.4. Multivariable Model for Histologic Grade (II–III vs. I)

The final grade model was fitted on 83/86 carcinomas; three cases were excluded due to missing data for the predictor “previous mammary tumours”. Intermediate/high grade (II–III) was more frequent in bitches with a previous history of mammary tumours (OR 9.95, 95% CI 2.56–38.68; *p* = 0.001) and in those with bloody nipple discharge (OR 70.34, 95% CI 3.34–1480.68; *p* = 0.006), and it was less frequent as the number of synchronous nodules increased (OR per additional nodule 0.56, 95% CI 0.36–0.88; *p* = 0.011) (Table 4). Because bloody discharge was rare, effect estimates for this predictor were statistically imprecise, as reflected by the wide confidence interval.

Model calibration was acceptable (slope 1.17; intercept 0.09), and discrimination was high (AUC 0.859, 95% CI 0.776–0.943). Because bloody discharge produced quasi-separation in standard logistic regression, a Hosmer–Lemeshow statistic was not estimable; penalised (Firth) estimates are therefore reported. The prediction equation and a worked example are provided in Supplementary Box S2.

### 3.5. Model Performance and Sensitivity Analyses

Both models demonstrated low multicollinearity (mean VIF 1.21 for malignancy; 1.01 for grade). Univariable screening results are reported in Tables S1 and S2. In a multiple-imputation sensitivity analysis restricted to the detection-to-surgery interval (delay_mo; 64/286 missing; m = 40), sensitivity models were fitted using logistic regression with dog-clustered standard errors. Effect directions were consistent with the primary complete-case penalised model: tumour size, and age at neutering remained clearly associated with malignancy, whereas estimates for reported rapid growth and the exploratory delay threshold were slightly attenuated but directionally consistent.

## 4. Discussion

In this retrospective study of 286 excised mammary nodules from 92 bitches with M0 mammary disease at presentation, two preoperative, nodule-level clinical prediction models were developed based exclusively on history and physical examination. The tumour size, owner-reported rapid growth, interval between detection and surgery, and reproductive history (age at neutering) were useful in anticipating a malignant (rather than benign/non-neoplastic) histological diagnosis. Among mammary carcinomas, a previous history of mammary tumours, bloody nipple discharge and fewer synchronous nodules detected on preoperative examination were informative in identifying intermediate/high histological grade (II–III). Following dog-level bootstrap internal validation, both models demonstrated good discrimination and acceptable calibration, suggesting their potential role in preoperative risk stratification and surgical decision making in routine practice. These models are intended to complement, rather than replace, histopathology and clinical judgement, and external validation is required before routine implementation.

The proportion of malignant histological diagnoses in our study population t was 30.4% (87/286 nodules). Reported malignancy proportions in canine mammary tumour studies vary widely and are highly sensitive to study population definition and study setting. This includes whether the denominator is defined at the dog or nodule level, whether non-neoplastic proliferative lesions are grouped with benign diagnoses and whether advanced presentations (e.g., metastatic or inflammatory disease) and/or referral pathology submissions are represented. As our study population was restricted to dogs with M0 disease at presentation and surgically excised lesions, absolute malignancy proportions should be compared cautiously across studies. In this context, individualised nodule-level risk estimates may be more informative than a single headline prevalence for guiding preoperative staging and surgical planning [1,4,17,20].

Tumour size remains a consistent clinical parameter associated with malignant histology, and our findings are consistent with those of previous studies [20,21]. However, tumour size alone should not be used to exclude malignancy. In our sample, 40.2% (35/87) of malignant lesions measured less than 1 cm, and the majority of malignant nodules were classified as T1 (less than 3 cm). In contrast, Sorenmo et al. (2009) reported that tumours measuring less than 1 cm were rarely malignant in their dataset, highlighting that the prevalence of very small malignant lesions can differ substantially across settings [22]. While our study cannot determine progression at the level of individual lesions, these results are consistent with a range of histological changes across mammary lesions. They also reinforce the importance of appropriately assessing and histopathologically evaluating even small, discrete nodules [2,15,22,23]. Ulceration was uncommon in our M0 sample and occurred in both benign and malignant lesions. Accordingly, the univariable association was imprecise (Table S1), and ulceration was not retained in the final multivariable model. Clinically, ulceration should be interpreted in context and may warrant thorough staging and histopathologic assessment, but its absence does not exclude malignancy.

Growth kinetics and time to surgery also carried clinically relevant information. The owner-reported rapid growth was associated with malignancy, as was a detection-to-surgery interval of more than 3.5 months, which is an exploratory ROC-derived threshold selected by maximising Youden’s index in our dataset. As this >3.5-month cut-off was derived in-sample, it should be considered hypothesis generating, may be sample-dependent, and requires external validation before it can be used as a decision threshold. Nevertheless, the direction of association is clinically coherent and supports the timely assessment of, and avoidance of unnecessary deferral in, cases where a discrete mammary lesion is detected, particularly when rapid growth is reported.

In this setting, reproductive history variables require cautious interpretation. In our malignancy model, later age at neutering was associated with lower odds of a malignant diagnosis, which is an unexpected finding given the commonly reported protective effect of early ovariohysterectomy on lifetime mammary tumour incidence [24,25]. This apparent discrepancy likely reflects the fact that our outcome concerns malignancy among excised lesions in dogs already presenting with mammary nodules, which is conceptually distinct from the effect of neutering timing on lifetime tumour incidence. Early neutering was uncommon in our dataset (≤3 years: 5/82 dogs with available age-at-neutering data), and the observed association is therefore best interpreted as study population-specific and potentially influenced by selection and residual confounding factors (including the indication and timing of neutering, intact status coding, and imperfect proxies of lifetime hormonal exposure). This finding should not be used to inform preventive recommendations. More broadly, our results are compatible with the concept that ovarian status may be linked not only to tumour occurrence but also to the distribution of lesions across a histological spectrum. However, we must acknowledge that causal inference is not possible with the present design [23]. In intact bitches presenting with mammary tumours, concurrent ovariohysterectomy is routinely considered when feasible for standard reproductive tract indications. However, evidence of an oncologic benefit of routine concurrent ovariohysterectomy in bitches with mammary carcinoma is scarce. Ovariohysterectomy performed at the time of excision of benign mammary tumors or hyperplastic lesions significantly lowers the risk of subsequent mammary tumor development in dogs [26]. Furthermore, in dogs with mammary carcinomas, ovariohysterectomy at the time of tumor removal does not provide a general survival benefit, but it reduces the risk of relapse in selected subgroups, particularly those with estrogen-receptor–positive or intermediate-grade tumors [26].

Among carcinomas, a prior history of mammary tumours was associated with intermediate/high histological grade, which is consistent with the possibility of prior neoplastic transformation and a potential mammary ‘field effect’ [22,27]. Bloody nipple discharge, although infrequent, was also associated with intermediate/high grade and improved discrimination in the carcinoma subset. Because this covariate was rare and produced quasi-separation in standard logistic regression, estimates relied on penalised methods and were accompanied by wide confidence intervals; confirmation in larger datasets is therefore advisable before considering this sign as a standalone risk indicator.

An inverse association was also observed between the number of synchronous nodules and intermediate/high-grade carcinoma, whereby bitches with fewer nodules at preoperative examination were more likely to harbour intermediate/high-grade disease. This pattern may reflect heterogeneity in multicentric presentations and/or case mix (for example, surgery prompted by a single clinically dominant nodule), and the present study design cannot distinguish between these explanations. From a clinical perspective, a solitary nodule that enlarges rapidly should be approached with at least the same level of concern as multifocal disease, and each lesion should be assessed individually.

Several considerations temper these findings. The retrospective, single-centre design may introduce selection and information biases, limiting generalisability. Some predictors, such as reported growth rate and the detection-to-surgery interval, relied on owner recall and may be misclassified. Complete-case analyses reduced the effective sample size for multivariable modelling. However, multiple-imputation sensitivity analyses restricted to the detection-to-surgery interval (m = 40) yielded estimates that were slightly attenuated but directionally consistent. This supports the robustness of the primary results to missingness in that predictor. Our inferences apply to surgically managed dogs with M0 mammary disease at presentation; metastatic disease (M1) and inflammatory mammary carcinoma were excluded by design. Accordingly, the proposed models should not be extrapolated to M1 disease or IMC presentations, and dedicated studies are warranted for these higher-risk clinical scenarios. Grade analyses were restricted to mammary carcinomas and accordingly excluded non-carcinoma malignant tumours (e.g., sarcomas and malignant myoepithelioma). Finally, histologic subtype is a post-excision variable and was therefore not considered a candidate predictor in models intended to rely exclusively on preoperative history and physical examination. Moreover, many subtypes are uncommon, and incorporating the subtype in a dataset of this size would likely result in sparse cells and unstable estimates. Future studies with larger, multicenter carcinoma study populations should evaluate whether the subtype adds incremental predictive value once robust preoperative proxies can be identified and validated [2,15,16].

Despite these limitations, the study benefited from standardised diagnostic procedures, surgical management and histopathology within a dedicated unit, the explicit handling of clustered data (nodules nested within dogs), penalised multivariable regression to reduce small-sample bias, and internal validation with calibration reporting. In day-to-day practice, these risk estimates can be combined with clinical judgement to support risk-stratified staging (including nodal assessment and thoracic imaging), guide surgical planning (including a consideration of lymph node assessment or sampling in higher-risk cases) and inform owner counselling. Ultrasonography may be used as an adjunct when additional information is sought; however, conventional B-mode and Doppler assessments show only moderate discriminatory performance, and elastography-based techniques, while promising in selected studies, are not routinely available and do not replace histopathology for definitive diagnosis [12,13].

## 5. Conclusions

In summary, within a clearly defined M0 study population, a small set of accessible preoperative findings was associated with malignancy status (malignant versus benign/non-neoplastic histology) and intermediate/high histological grade among carcinomas. When used alongside clinical expertise, these estimates could help to prioritise staging and inform surgical planning. Ideally, external, multicentre validation in prospective studies with standardised imaging and pathology will determine the estimates’ readiness for routine implementation and further clarify the contribution of potentially modifiable factors, including time to surgery and reproductive variables, to preoperative risk assessment.

## Figures and Tables

**Table 1:** Epidemiologic and clinical characteristics of 92 bitches with M0 mammary disease (local or regional) at presentation that contributed 286 surgically excised mammary nodules.

Variable	Category/Statistic	*n*/N (%) or Mean ± SD (Median, IQR)
Age (years)	—	10.1 ± 2.6 (9.9, 8.6–12.2)
Body weight (kg)	—	14.3 ± 10.6 (10.1, 5.7–22.0)
Age at neutering (years) *	—	9.0 ± 2.9 (9.0, 7–11)
Number of synchronous mammary nodules at presentation	—	2.91 ± 2.26 (2.0, 1–4)
Bitches with ≥2 synchronous nodules at presentation ^α^	Yes	63/92 (68.5%)
Clinical stage at presentation ^†^	Local disease	81/92 (88.0%)
	Regional disease	11/92 (12.0%)
Breed	Purebred	67/92 (72.8%)
	Crossbred	25/92 (27.2%)
Reproductive status at surgery	Intact	29/92 (31.5%)
	Spayed	63/92 (68.5%)
Spayed due to pyometra	Yes	7/92 (7.6%)
History of pseudopregnancy ^‡^	Yes	25/69 (36.2%)
Previous mammary tumours ^§^	Yes	15/85 (17.6%)
Previous mammary tumour biopsy ^¶^	Yes	13/15 (86.7%)
History of litters ^‖^	Yes	9/53 (17.0%)
Previous pharmacologic treatment	Yes	24/92 (26.1%)
Previous cabergoline treatment	Yes	14/92 (15.2%)
Palpable inguinal lymph node	Yes	17/92 (18.5%)
Palpable axillary lymph node	Yes	5/92 (5.4%)
Inguinal lymph node metastasis	Yes	9/92 (9.8%)
Axillary lymph node metastasis	Yes	0/92 (0.0%)

* Age at neutering available for 82/92 dogs (10 missing). ^α^ Synchronous nodules were defined as the number of mammary nodules detected on preoperative mammary examination at first presentation. ^†^ Clinical stage defined according to the modified WHO TNM system for canine mammary tumours; regional disease denotes M0 with histologically or cytologically confirmed regional lymph node metastasis. ^‡^ Pseudopregnancy data available for 69/92 dogs (23 missing). ^§^ Previous mammary tumours recorded for 85/92 dogs (7 missing). ^¶^ Previous mammary tumour biopsy refers to histopathological confirmation among dogs with a history of previous mammary tumours (available for 15 dogs). ^‖^ History of litters available for 53/92 dogs (39 missing). Abbreviations: IQR, interquartile range; —, not applicable.

**Table 2:** Clinical and histopathologic characteristics of 286 surgically excised mammary nodules from 92 bitches with M0 mammary disease at presentation.

Variable	Category/Statistic	*n*/N (%) or Median (IQR)
Mammary secretion *	Non-sanguineous (none/serous/milky/other non-bloody)	273/286 (95.5%)
	Sanguineous	13/286 (4.5%)
Signs of inflammation	Absent	222/286 (77.6%)
	Present	64/286 (22.4%)
Adherence to deep tissues	Absent	279/286 (97.6%)
	Present	7/286 (2.4%)
Ulceration	Absent	276/286 (96.5%)
	Present	10/286 (3.5%)
Maximum tumour size (cm)	—	0.6 (0.3–1.6); mean 1.34 ± 1.91
Malignancy status	Benign	199/286 (69.6%)
	Malignant	87/286 (30.4%)
Histological grade ^†^ (malignant carcinomas only, *n* = 86)	Grade I	49/86 (57.0%)
	Grade II	20/86 (23.3%)
	Grade III	17/86 (19.8%)

* Categories reconstructed from clinical records and harmonised to avoid overlap. For modelling, secretion was recoded as a binary variable (sanguineous vs. other/none). ^†^ Grading was performed in malignant carcinomas only; one malignant myoepithelioma (grade II) was identified and excluded from grade analyses. Abbreviations: IQR, interquartile range; —, not applicable.

**Table 3:** Multivariable Firth penalised logistic regression model predicting a malignant histologic diagnosis in mammary nodules from bitches with M0 mammary disease at presentation (complete-case analysis: 153/286 nodules).

Variable	OR (95% CI)	Coefficient (B)	*p* Value
Constant (intercept)	—	−0.830	—
Age at neutering (years)	0.76 (0.65–0.88)	−0.274	<0.001
Maximum tumour size (cm)	1.46 (1.17–1.82)	0.378	0.001
Rapid growth (yes vs. no)	3.11 (1.15–8.41)	1.135	0.025
Delay > 3.5 months (yes vs. no)	3.20 (1.23–8.27)	1.163	0.017

Model performance: AUC = 0.805 (95% CI 0.731–0.880); calibration slope = 1.06, intercept = 0.03; Hosmer–Lemeshow χ^2^ = 4.68, *p* = 0.79. Internal validation: 1000 dog-level bootstrap replicates; optimism-corrected AUC = 0.799. Notes: Firth logistic regression. Coefficients (B) are log-odds (approximately ln[OR]) and are rounded to three decimals. The intercept OR is not interpretable; no *p* value is reported for the constant. Abbreviations: OR, odds ratio; CI, confidence interval; AUC, area under the receiver operating characteristic curve.

**Table 4:** Multivariable Firth penalised logistic regression model predicting intermediate/high histologic grade (II–III vs. I) among mammary carcinomas from bitches with M0 mammary disease at presentation (complete-case analysis: 83/86 carcinomas).

Variable	OR (95% CI)	Coefficient (B, Log-Odds)	*p* Value
Constant (intercept)	—	−0.28	— ^‡^
Previous mammary tumours (yes vs. no)	9.95 (2.56–38.68)	2.30	0.001
Sanguineous secretion (yes vs. no)	70.34 (3.34–1480.68)	4.25	0.006
Number of nodules (per unit increase)	0.56 (0.36–0.88)	−0.58	0.011

Model performance: AUC = 0.859 (95% CI 0.776–0.943); calibration slope = 1.17, intercept = 0.09. Goodness of fit: Hosmer–Lemeshow test not estimable due to quasi-separation in standard logistic regression. Notes: Firth logistic regression; wide CIs reflect sparse data for sanguineous secretion. Model fitted in 83/86 carcinomas (3 excluded due to missing data for “previous mammary tumours”). Continuous predictor modelled per unit increase (number of synchronous nodules). ^‡^ Intercept reported to enable model reproduction; an odds ratio for the intercept is not interpretable. Abbreviations: OR, odds ratio; CI, confidence interval; AUC, area under the receiver operating characteristic curve.

## Data Availability

The datasets generated and/or analysed during the current study are not publicly available due to their origin from confidential clinical records, but de-identified data and the statistical analysis code are available from the corresponding author upon reasonable request subject to institutional approval and data protection requirements.

## Supplementary Materials

The following supporting information can be downloaded at https://www.mdpi.com/article/10.3390/ani16030421/s1. Table S1. Univariable logistic regression for malignant histologic diagnosis (malignant vs. benign/non-neoplastic) in 286 mammary nodules from 92 bitches with M0 mammary disease at presentation (standard errors clustered by dog). Table S2. Univariable logistic regression for intermediate/high histologic grade (II–III vs. I) among 86 mammary carcinomas from bitches with M0 mammary disease at presentation (standard errors clustered by dog). Table S3. Proportion of malignant histologic diagnoses by modified WHO TNM tumour size category (Rutteman T component) in 286 surgically excised canine mammary nodules. Figure S1. Maximum tumour diameter in benign versus malignant canine mammary nodules (n = 286 nodules from 92 bitches with M0 mammary disease at presentation). Boxplot comparing maximum tumour diameter between benign/non-neoplastic (n = 199) and malignant (n = 87) mammary nodules. Malignant nodules were larger (median 1.3 cm [IQR 0.5–3.1]) than benign/non-neoplastic nodules (median 0.5 cm [IQR 0.3–1.0]) (Mann–Whitney U test, z = −5.54, *p* < 0.001). Notably, 40.2% (35/87) of malignant nodules measured <1 cm, indicating that small size alone does not preclude malignancy. Abbreviations: IQR, interquartile range. Figure S2. Maximum tumour diameter by histologic grade among canine mammary carcinomas (n = 86). Boxplot comparing maximum tumour diameter in grade I carcinomas (n = 49; median 1.0 cm [IQR 0.5–2.3]) versus grade II–III carcinomas (n = 37; median 1.6 cm [IQR 0.8–3.6]). No statistically significant difference was observed (Mann–Whitney U test, z = −1.82, *p* = 0.069). Abbreviations: IQR, interquartile range. Figure S3. Proportion of malignant histologic diagnoses by modified WHO TNM tumour size category (Rutteman T component) in 286 surgically excised canine mammary nodules. Bars represent the proportion of malignant lesions within each size category (T1 <3 cm; T2 3–5 cm; T3 >5 cm). Despite the size gradient, most malignant nodules in the sample were classified as T1 (<3 cm). Box S1. Clinical calculator for malignant histologic diagnosis in canine mammary nodules (Firth logistic regression; complete-case analysis, n = 153 nodules). Box S2. Clinical calculator for intermediate/high histologic grade (II–III vs. I) among canine mammary carcinomas (Firth logistic regression; complete-case analysis, n = 83 carcinomas).

## Abbreviations

The following abbreviations are used in this manuscript:

CMT	Canine mammary tumour
AUC	Area under the curve
VTH	Veterinary Teaching Hospital
OR	Odds ratio
