# Supplementary material for: Preoperative Clinical Predictors of Histologic Malignancy and Carcinoma Grade in 286 Canine Mammary Nodules from 92 Bitches: A Retrospective Study
Source: Animals (Basel). 2026 Jan 29;16(3):421. doi: 10.3390/ani16030421 (PMC12896456; doi:10.3390/ani16030421)
Supplement: Supplementary file 1 [file animals-16-00421-s001.zip › animals-4095250-supplementary.pdf]

Supplementary

Table S1. Univariable logistic regression for malignant histologic diagnosis (malignant vs. benign/non-neoplastic) in 286 mammary nodules from 92 bitches with M0 mammary disease at presentation (standard errors clustered by dog).

**Variable**	**OR (95% CI)**	***p* Value**
Age (years)	0.98 (0.84–1.14)	0.762
Purebred	1.06 (0.53–2.15)	0.868
Body condition score	0.83 (0.57–1.19)	0.310
Body weight (kg)	1.04 (1.00–1.07)	0.030
Spayed at surgery	1.85 (0.93–3.67)	0.079
Spayed due to pyometra	1.22 (0.43–3.45)	0.702
Age at neutering (years)	0.82 (0.73–0.92)	0.001
Previous mammary tumours	2.20 (0.84–5.79)	0.110
Rapid tumour growth	1.91 (0.79–4.67)	0.153
Mammary secretion: lacteal vs. none	0.24 (0.07–0.84)	0.025
Mammary secretion: sanguineous vs. none/other	2.82 (0.89–8.86)	0.077
Inflammation at nodule site	1.81 (1.05–3.10)	0.032
Ulceration	2.37 (0.40–13.94)	0.341
Maximum tumour size (cm)	1.64 (1.31–2.05)	<0.001
Number of nodules	0.93 (0.77–1.14)	0.498
Palpable inguinal lymph node	3.40 (1.67–6.95)	0.001
Hematology: neutrophils (bands, %)	1.20 (1.09–1.33)	<0.001
Hematology: lymphocytes (absolute count)	1.04 (1.02–1.06)	<0.001
Hematology: neutrophils (bands, absolute count)	3.05 (1.48–6.27)	0.002

Notes: Univariable logistic regression with dog-level clustered standard errors (vce[cluster dog]). OR > 1 indicates greater odds of malignancy. Only variables with $p < 0.15$ or clear a priori clinical relevance are shown.

Table S2. Univariable logistic regression for intermediate/high histologic grade (II–III vs. I) among 86 mammary carcinomas from bitches with M0 mammary disease at presentation (standard errors clustered by dog).

**Variable**	**OR (95% CI)**	***p* Value**
Number of nodules	0.66 (0.51–0.86)	0.002
Mammary secretion: sanguineous vs. none	7.40 (1.81–30.33)	0.005
Serum creatinine	15.30 (2.11–110.85)	0.007
Mammary secretion: any recorded vs. none	15.43 (1.59–149.32)	0.018
Previous mammary tumours	5.02 (1.24–20.27)	0.024
Monocytes (absolute count)	0.19 (0.04–0.87)	0.032
White blood cells	0.86 (0.75–0.99)	0.038
Time since detection (days)	0.94 (0.89–1.00)	0.038
Serum glucose	0.95 (0.91–1.00)	0.041
Tumour size ≥ 1 cm	3.44 (2.06–5.75)	<0.001

Notes: Univariable logistic regression with dog-level clustered standard errors (vce[cluster dog]). OR > 1 indicates greater odds of intermediate/high grade. "Mammary secretion: any recorded vs. none" reflects the presence of any mammary secretion noted in the clinical record, irrespective of its specific appearance.

Table S3. Proportion of malignant histologic diagnoses by modified WHO TNM tumour size category (Rutteman T component) in 286 surgically excised canine mammary nodules.

Rutteman T Category (Maximum Diameter)	Total Nodules, n (%)	Malignant Nodules, n/N (%)	Malignancy Proportion (95% CI) *
T1 (<3 cm)	250 (87.4)	62/250 (24.8)	24.8% (19.6–30.6)
T2 (3–5 cm)	22 (7.7)	13/22 (59.1)	59.1% (36.4–79.3)
T3 (>5 cm)	14 (4.9)	12/14 (85.7)	85.7% (57.2–98.2)
Total	286 (100)	87/286 (30.4)	30.4% (25.1–36.1) †

* Exact binomial 95% confidence intervals. † Total CI calculated from 87/286.

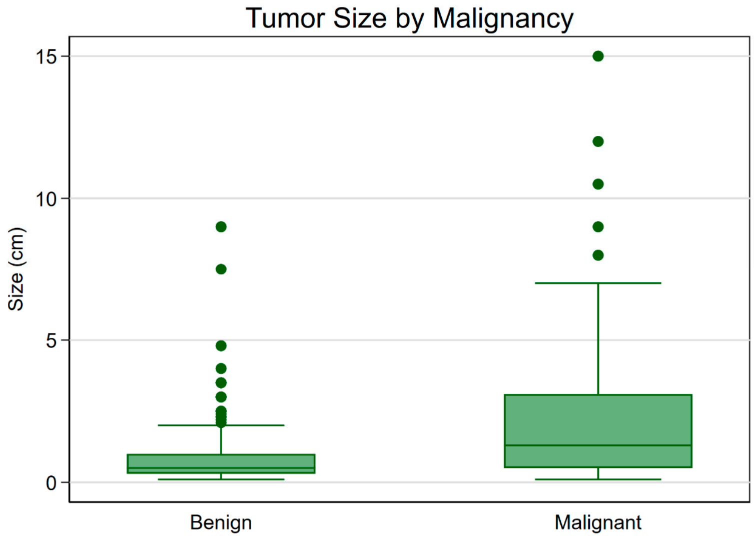

Figure S1. Maximum tumour diameter in benign versus malignant canine mammary nodules (n = 286 nodules from 92 bitches with M0 mammary disease at presentation). Boxplot comparing maximum tumour diameter between benign/non-neoplastic (n = 199) and malignant (n = 87) mammary nodules. Malignant nodules were larger (median 1.3 cm [IQR 0.5–3.1]) than benign/non-neoplastic nodules (median 0.5 cm [IQR 0.3–1.0]) (Mann–Whitney U test, z = −5.54, $p < 0.001$). Notably, 40.2% (35/87) of malignant nodules measured <1 cm, indicating that small size alone does not preclude malignancy. Abbreviations: IQR, interquartile range.

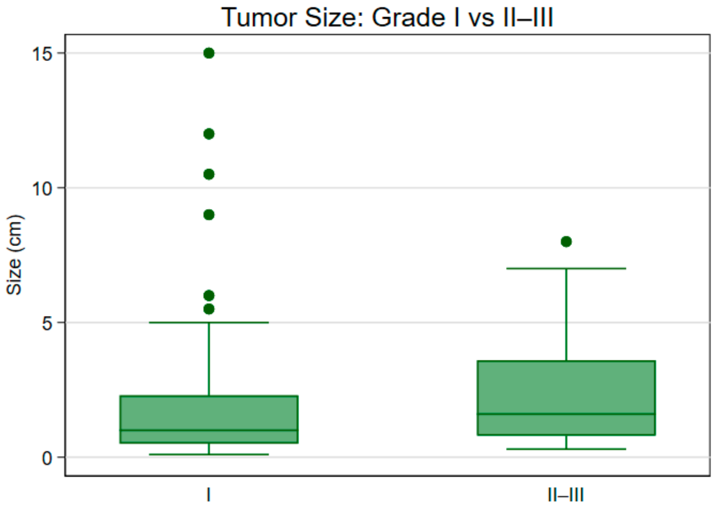

Figure S2. Maximum tumour diameter by histologic grade among canine mammary carcinomas (n = 86). Boxplot comparing maximum tumour diameter in grade I carcinomas (n = 49; median 1.0 cm [IQR 0.5–2.3]) versus grade II–III carcinomas (n = 37; median 1.6 cm [IQR 0.8–3.6]). No statistically significant difference was observed (Mann–Whitney U test, z = −1.82, $p = 0.069$). Abbreviations: IQR, interquartile range.

https://doi.org/10.3390/xxxxx

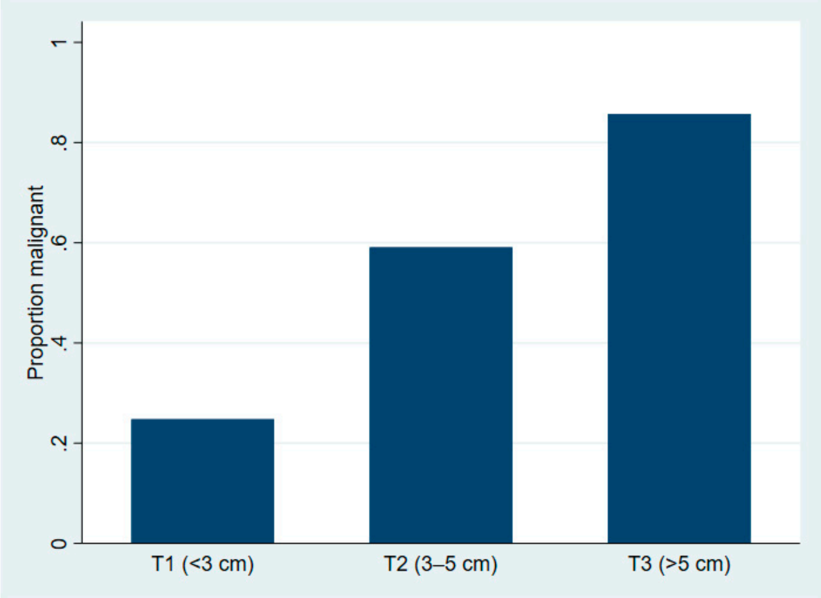

Figure S3. Proportion of malignant histologic diagnoses by modified WHO TNM tumour size category (Rutteman T component) in 286 surgically excised canine mammary nodules. Bars represent the proportion of malignant lesions within each size category (T1 <3 cm; T2 3–5 cm; T3 >5 cm). Despite the size gradient, most malignant nodules in the sample were classified as T1 (<3 cm).

Supplementary Box S1.

Clinical calculator for malignant histologic diagnosis in canine mammary nodules (Firth logistic regression; complete-case analysis, n = 153 nodules).

The malignancy model was developed using Firth's penalised logistic regression in 153 mammary nodules with complete data. The outcome was histological malignancy (malignant vs. benign/non-neoplastic) at the nodule level. The final model included four routinely available preoperative variables: age at neutering (years; intact bitches coded as 0), maximum tumour size (cm), rapid growth (yes vs. no) and a detection-to-surgery interval longer than 3.5 months (yes vs. no). The 3.5-month threshold for the detection-to-surgery interval was derived from ROC analysis using Youden's index and then dichotomised for modelling.

For each nodule, let the following apply:

– AgeN = age at neutering in years (0 if intact);
– Size = maximum tumour diameter in cm;
– Rapid = 1 if rapid growth is reported, 0 otherwise;
– Delay = 1 if the time from detection to surgery is >3.5 months, 0 otherwise.

The model's linear predictor (η) is:

$$\eta = \beta_0 + \beta_1 \cdot \text{AgeN} + \beta_2 \cdot \text{Size} + \beta_3 \cdot \text{Rapid} + \beta_4 \cdot \text{Delay},$$

where β_0–β_4 are the regression coefficients reported in Table 3. The predicted probability of malignancy (p) is then obtained via the logistic transformation:

$$p = 1/(1 + \exp(-\eta)).$$

Worked example

As an illustration, consider a bitch neutered at 9 years of age with a 2.5 cm mammary nodule showing rapid growth and a delay to surgery greater than 3.5 months (AgeN = 9, Size = 2.5, Rapid = 1, Delay = 1). Substituting these values and the coefficients from Table 3 into the equation above yields an estimated probability of malignancy of approximately 0.49 (49%).

https://doi.org/10.3390/xxxxx

Supplementary Box S2.

Clinical calculator for intermediate/high histologic grade (II–III vs. I) among canine mammary carcinomas (Firth logistic regression; complete-case analysis, n = 83 carcinomas).

A separate Firth's penalised logistic regression model was constructed in 83 malignant epithelial tumours (carcinomas) with complete data to predict high histological grade, which is defined as grades II–III versus grade I. The final model included three preoperative predictors: previous history of mammary tumours (yes vs. no), sanguineous nipple discharge (yes vs. no) and the number of synchronous nodules (continuous). These variables are routinely obtainable at presentation and reflect both the dog's history and the clinical appearance of the mammary chain.

For a given carcinoma, let the following apply:

– Prev = 1 if a previous history of mammary tumours is recorded (0 otherwise);
– Sang = 1 if sanguineous nipple discharge is present (0 otherwise);
– Nodules = total number of synchronous mammary nodules.

The model's linear predictor (η) is

$$\eta = \beta_0 + \beta_1 \cdot \text{Prev} + \beta_2 \cdot \text{Sang} + \beta_3 \cdot \text{Nodules},$$

where β_0–β_3 are the regression coefficients reported in Table 4. The predicted probability of high-grade carcinoma (p) is then

$$p = 1/(1 + \exp(-\eta)).$$

Worked example

For example, a carcinoma in a bitch with a documented history of previous mammary tumours, sanguineous discharge from the affected mammary gland and two synchronous nodules (Prev = 1, Sang = 1, Nodules = 2) would, using the coefficients in Table 4, have an estimated probability of high histological grade of approximately 0.99 (99%).

An important caveat concerns the variable "sanguineous nipple discharge". Although inclusion of this predictor improved the model's discriminative ability and it is biologically plausible that bloody discharge marks aggressive disease, this sign was present in only a small number of cases. Consequently, the odds ratio for sanguineous discharge is associated with an extremely wide 95% confidence interval (3.34–1480.68), reflecting substantial statistical imprecision rather than a precise quantitative effect. In practice, sanguineous discharge should therefore be interpreted as a strong qualitative warning sign within the context of the full model and the broader clinical picture and not as a stand-alone quantitative prediction. Further confirmation in larger, independent studies is warranted before the formal incorporation of this predictor into routine risk algorithms.

https://doi.org/10.3390/xxxxx
